# Harnessing advances in computer simulation to inform policy and planning to reduce alcohol-related harms

**DOI:** 10.1007/s00038-017-1041-y

**Published:** 2017-10-19

**Authors:** Jo-An Atkinson, Dylan Knowles, John Wiggers, Michael Livingston, Robin Room, Ante Prodan, Geoff McDonnell, Eloise O’Donnell, Sandra Jones, Paul S. Haber, David Muscatello, Nadine Ezard, Nghi Phung, Louise Freebairn, Devon Indig, Lucie Rychetnik, Jaithri Ananthapavan, Sonia Wutzke, On behalf of the alcohol modelling consortium, Jo-An Atkinson, Dylan Knowles, John Wiggers, Robin Room, Michael Livingston, Kate Conigrave, Chris Rissel, Philip Coates, David Muscatello, Paul Haber, Nghi Phung, Ralph Moore, Leena Gupta, Nadine Ezard, Fiona Renshaw, Sandra Jones, Karen Price, Jo Mitchell, Genevieve Whitlam, Nicola Lewis, Ante Prodan, Eloise O’Donnell, Geoff McDonnell, Mark Heffernan, Louise Freebairn, Michael Lambert, Jaithri Ananthapavan, Lucie Rychetnik, Sally Redman, Alan Shiell, Devon Indig, Luke Penza, Sonia Wutzke, Andrew Wilson

**Affiliations:** 1 Decision Analytics, Sax Institute, Sydney, Australia; 2 The Australian Prevention Partnership Centre, Sax Institute, Ultimo, NSW Australia; 3 Sydney Medical School, University of Sydney, Camperdown, Australia; 4 Anthrodynamics Simulation Services, Saskatoon, Canada; 5 Hunter New England Population Health, Newcastle, NSW Australia; 6 School of Medicine and Public Health, University of Newcastle, Callaghan, NSW Australia; 7 Centre for Alcohol Policy Research, La Trobe University, Melbourne, Australia; 8 Department of Clinical Neuroscience, Karolinska Institutet, Stockholm, Sweden; 9 Centre for Social Research on Alcohol and Drugs, Stockholm University, Sveaplan, Stockholm, Sweden; 10 School of Computing, Engineering and Mathematics, Western Sydney University, Parramattu, Australia; 11 Centre for Health and Social Research, Australian Catholic University, Melbourne, Australia; 12 Drug Health Services, Royal Prince Alfred Hospital, Sydney, Australia; 13 Faculty of Medicine, University of NSW, Sydney, Australia; 14 Alcohol and Drug Service, St Vincent’s Hospital, Sydney, Australia; 15 Drug Health Western Sydney Local Health District, Sydney, Australia; 16 Liver Addiction Research Unit, Storr Liver Centre, Westmead Institute of Medical Research, Sydney, Australia; 17 Knowledge Translation and Health Outcomes, Epidemiology Section, ACT Health, Canberra, Australia; 18 School of Medicine Sydney, University of Notre Dame Australia, Sydney, Australia; 19 School of Public Health, University of Sydney, Camperdown, Australia; 20 Faculty of Health, Deakin Health Economics, Centre for Population Health Research, Deakin University, Burwood, Australia; 21 Menzies Centre for Health Policy, University of Sydney, Sydney, Australia

**Keywords:** Agent-based modelling, Alcohol-related harm, Prevention policy, Evidence synthesis

## Abstract

**Objectives:**

Alcohol misuse is a complex systemic problem. The aim of this study was to explore the feasibility of using a transparent and participatory agent-based modelling approach to develop a robust decision support tool to test alcohol policy scenarios before they are implemented in the real world.

**Methods:**

A consortium of Australia’s leading alcohol experts was engaged to collaboratively develop an agent-based model of alcohol consumption behaviour and related harms. As a case study, four policy scenarios were examined.

**Results:**

A 19.5 ± 2.5% reduction in acute alcohol-related harms was estimated with the implementation of a 3 a.m. licensed venue closing time plus 1 a.m. lockout; and a 9 ± 2.6% reduction in incidence was estimated with expansion of treatment services to reach 20% of heavy drinkers. Combining the two scenarios produced a 33.3 ± 2.7% reduction in the incidence of acute alcohol-related harms, suggesting a synergistic effect.

**Conclusions:**

This study demonstrates the feasibility of participatory development of a contextually relevant computer simulation model of alcohol-related harms and highlights the value of the approach in identifying potential policy responses that best leverage limited resources.

**Electronic supplementary material:**

The online version of this article (doi:10.1007/s00038-017-1041-y) contains supplementary material, which is available to authorized users.

## Introduction

Alcohol misuse is a complex systemic problem. Globally, alcohol is estimated to result in 3.3 million deaths each year, with resultant health and social costs accounting for more than 1% of the gross national product of high- and middle-income countries (Rehm et al. 2009; World Health Organization 2014). Evidence suggests that alcohol misuse and related harms arise from a complex aetiology that includes a range of interacting individual, sociocultural, economic and environmental risk factors (International Center for Alcohol Policies 2009). A number of options are available for addressing the harms arising from alcohol misuse including pricing and taxation policies, regulating the availability of alcohol, modifying the alcohol consumption environment, drink-driving countermeasures, restrictions on marketing of alcohol products, education and persuasion, clinical treatment and early interventions (Babor et al. 2010; Martineau et al. 2013). Despite the availability of evidence regarding the effectiveness of such options, the selection of effective responses to this complex problem is challenged by a lack of clarity on how multi-level risk factors of alcohol misuse interact and change over time. There is also uncertainty regarding the impacts of interventions at the population level in particular contexts and the likely effects of combining them, which is further compounded by differing stakeholder priorities and views regarding the most appropriate approaches. Further, political considerations, community advocacy and industry lobbying can give rise to promulgation of, or resistance to, particular policy response options.

To better understand complexity and manage uncertainty, a range of sciences make use of computer simulation to estimate the impacts of certain actions (Winsberg 2010). Agent-based modelling is one type of computer simulation modelling approach that has been used in this way since the mid-1990s to understand social dynamics (Epstein and Axtell 1996). More recently, it has been applied to complex public health problems to help inform policy and practice (Nianogo and Arah 2015). Agent-based modelling involves the development of an artificial population of individuals (agents) with key characteristics of a real-world population and simple behavioural rules to define their activities and interactions within a given environment and with each other (Epstein and Axtell 1996). Agent interactions and introduction of changes or interventions to the environment result in emergent behaviours and outcomes at the population level, which are plotted over time. Unlike other forecast modelling, agent-based models explicitly represent the causal hypothesis underlying a complex problem, accounting for interdependencies of risk factors, changes over time, feedback loops (vicious and virtuous cycles) and real-world inertia and delay. Agent-based model development draws together into a single, coherent representation a variety of evidence sources such as conceptual models, research evidence and routine data. To establish the basic plausibility of the articulated hypothesis, and ensure the resulting model is a valid representation of the real-world system, model outputs are compared against real-world historic data patterns across a range of indicators. The final product is a ‘what-if’ tool that can simulate different scenarios to explore their likely impacts over the short and longer term, before they are implemented in the real world.

Recent advances in software capability and more user-friendly interfaces mean that agent-based modelling is now more accessible to non-modellers. The improvements in model transparency that these software advances afford facilitates meaningful critique of model structure, parameterisation and assumptions by stakeholders, and builds greater confidence in the results produced by the model (Atkinson et al. 2017; Hovmand et al. 2014). This has allowed stakeholder engagement and participatory approaches to be embedded into the development of sophisticated simulation tools, which may address some of the challenges of building consensus for effective, coordinated action to address alcohol-related harms (Atkinson et al. 2017; Haute Autorite de Sante 2010; Hovmand et al. 2014).

Several agent-based models of alcohol consumption behaviour have been developed. They capture elements of the social dynamics and environmental influences on alcohol consumption behaviours, neurobiological responses to alcohol use, the evolution of alcohol outlets and alcohol consumers in a community, and the acute harms that arise from heavy alcohol consumption among young people (Fitzpatrick and Martinez 2012; Gorman et al. 2006; Lamy et al. 2011; Scott et al. 2016). These models provide valuable insights into alcohol consumption behaviour and harms, but the range of harms explored by each model, and the range of interventions able to be tested, are limited by model scope. In addition, these models were developed by researchers in isolation from stakeholders and decision makers. The aim of this study was to explore the feasibility of a participatory agent-based modelling approach that provides decision makers and key stakeholders with a sophisticated and robust decision support tool to test the likely impacts of different policy scenarios before they are implemented in the real world.

## Method

### Model development

The model development process drew on best practice guidelines for computational modelling and included grounding of assumptions in theory and evidence, sensitivity testing and calibration (Hammond 2015). The model was built using a participatory approach (Atkinson et al. 2017) that engaged a consortium of academics, policy experts, clinicians, programme planners and health economists in the process. A full description of the participatory approach and insights are provided elsewhere (Atkinson et al. 2017; Freebairn et al. 2017). The agent-based model was constructed using AnyLogic simulation software (http://www.anylogic.com/).

### Model purpose

The purpose of the model was to inform the choice and design of policy responses to the problem of alcohol-related harms in New South Wales, Australia, through the testing of combinations of interventions. Interventions and harms included in the model are listed in Online Resource 1—Boxes 1 and 2.

### Model environment

The model included features of the alcohol consumption environment in NSW, consisting of licensed venues (bars, pubs, nightclubs) grouped to represent entertainment precincts of varying density where individuals can consume alcohol. The model also contained work places, bottle shops (retail outlets) where alcohol can be purchased and homes where alcohol can be consumed. Bottle shops, workplaces and homes were distributed randomly within the environment, using variation in average travel time as a replacement for distance to bottle shops and licensed venues to simplify geographic and routing concerns and as a proxy for urban/rural differences. Licensed venues in the model were grouped into precincts to represent alcohol consumption destinations. Individuals in the model consume alcohol at licensed venues (bars, pubs, nightclubs), peer events (parties) and home. Individuals can be refused entry to, or ejected from, licensed venues due to time of day (i.e. closing time), identified intoxication (i.e. refusal to serve alcohol due to responsible service of alcohol (RSA) requirements) and ‘lockouts’ (a policy whereby new patrons are no longer admitted to venues after a particular time of night). Peer events are periodically hosted by individuals at home and are attended by members of an individual’s friend network. In the model, alcohol can be obtained from bottle shops for consumption at home, peer events and for pre-loading purposes (the practice of consuming alcohol at a private residence before going to a place where alcohol access might be expensive, limited or prohibited).

### Population attributes

**Fig. 1:**
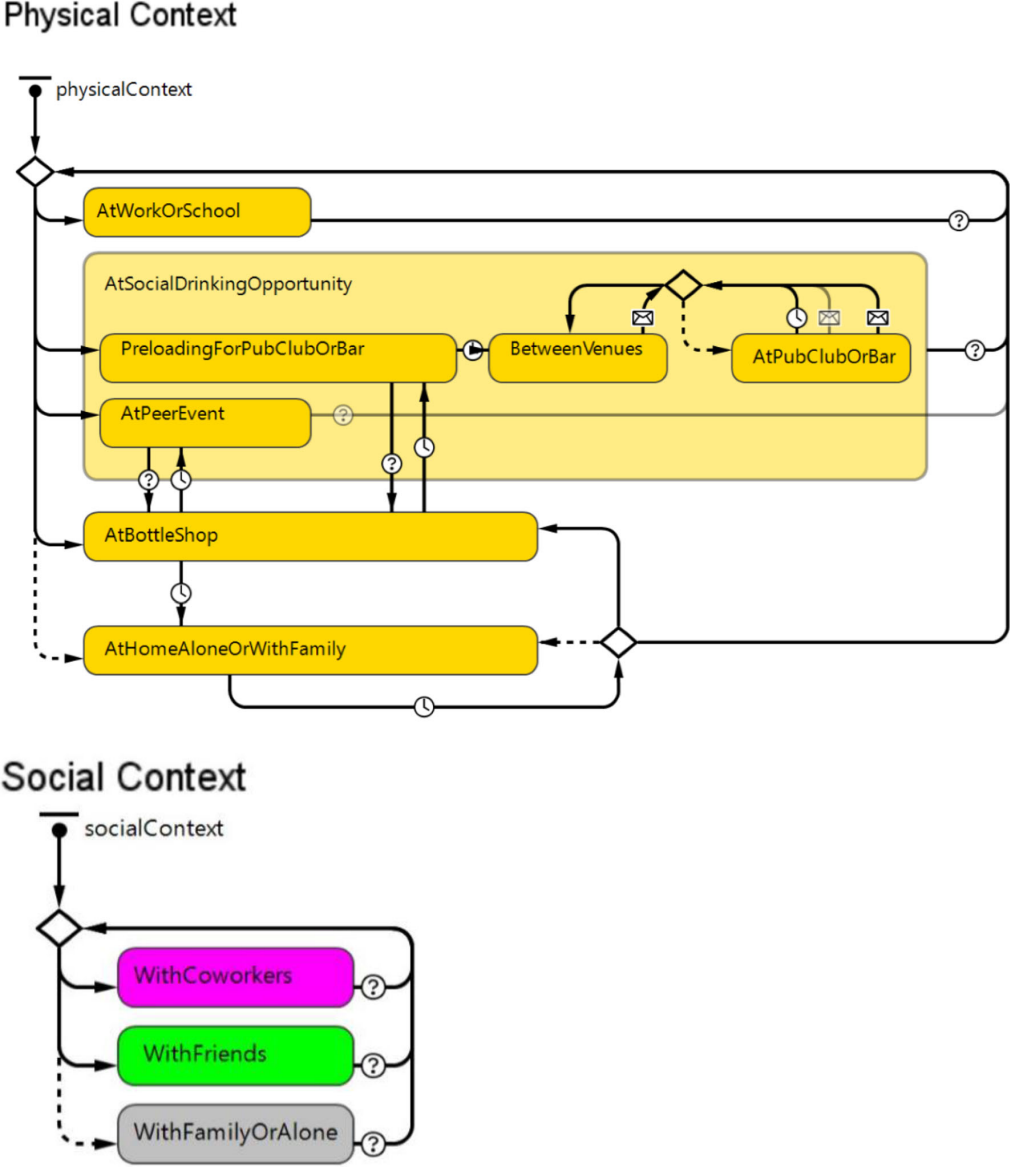
Physical and social contexts that influence an individual’s opportunities and motivation regarding alcohol consumption (NSW, Australia)

The model was initialised with a population of approximately 3.6 million individuals, representing approximately 75% of the adult population of NSW in 2011. At model initialisation, the distribution of demographic characteristics (age and sex), weight (in kilograms) and alcohol consumption (classified as low, moderate or heavy) reflected the empirical distribution of these factors in the NSW population for 2011. ‘Low’ alcohol consumption was defined as ≤ 2 standard drinks per day, ‘moderate’ was defined as 3–6 standard drinks per day and ‘heavy’ defined as ≥ 7 standard drinks per day (Marsden Jacob Associates 2012; National Health and Medical Research Council (NHMRC) 2009). Within the model, individuals age, interact with each other, consume alcohol and change habits over time. This generated alcohol risk behaviours, harms and statistics that can be used to compare intervention efficacy. For computational efficiency, the model population was divided into ‘representative’ and ‘synthetic’ persons. Representative persons are a population subset used much like a representative survey sample is used to extrapolate population-wide behaviours and statistics. Representative persons were modelled in detail and used to infer the behaviours and health outcomes of the total group of synthetic persons. Synthetic persons, modelled in significantly greater numbers due to their relative simplicity, acquire harms, are hospitalised, admitted to emergency, and/or die based on the activities and risk profile of their corresponding representative individual. Individuals in the model were situated in physical contexts (at home, work/study, licensed venues, bottle shops and peer events/home parties) and social contexts (alone, with friends or with co-workers) that change over time and influence alcohol consumption behaviours (Fig. 1).

### Alcohol consumption behaviour

Consistent with the COM-B conceptual model of alcohol consumption behaviour in context (Fishbein et al. 2001; Michie et al. 2011), a set of rules was established to govern the likelihood that an individual in the model will consume alcohol. These rules were grouped using the COM-B framework (Online Resource 1—Box 3) under: ‘Capacity’ which defines whether the individual can consume alcohol in their current state; ‘Opportunity’ defines where they might consume alcohol; and ‘Motivation’ defines how much they will consume alcohol once there, which over time resulted in an emergent alcohol consumption pattern (‘Behaviour’) (Online Resource 1—Box 4).

### Alcohol consumption episode and blood alcohol concentration (BAC)

**Fig. 2:**
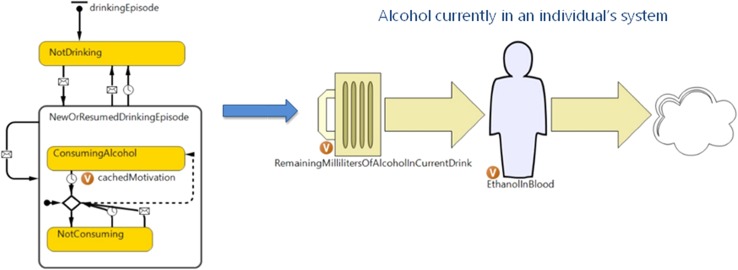
Representation of alcohol consumption episodes and blood alcohol concentration in continuous time (NSW, Australia)

For each individual in the model, the rules established under the COM-B framework influenced the likelihood of alcohol consumption at a given time. In the model, an individual’s BAC increased as alcohol was consumed and decreased as alcohol was metabolised by the body (Fig. 2). Using the sex and weight characteristics of the agent, the model used the Widmark equation (Widmark 1981) to estimate BAC in continuous time.

### Harm generation

Life history, demographics, alcohol consumption profile (daily home consumption vs periodic heavy binges) and context are rarely all captured in risk of harm estimations and harm reporting. To correctly reproduce harm levels using an individual-based model, the following were hypothesised:

1. The number of years spent consuming alcohol at a given level impacts risk of chronic harms.
2. Context affects acute harm generation. For example, road traffic accidents are only possible when an individual is in transit.
3. Demographics influence harm generation and outcomes. For example, males are more likely than females to engage in violence and are more likely to do so with other males; young adults are more likely to suffer acute harms than older adults.
4. Harms produce unequal health-care system burden. For example, violence/assaults rarely translate into hospitalisation; alcohol poisoning frequently results in emergency department (ED) presentations; acute expressions of severe chronic alcohol use disorder almost always produce hospitalisations.

*Acute harms* In the model, the risk of acute harms increases non-linearly with BAC, age, sex and context at any given point in time (see Online Resource 2 for risk estimates). Acute harms may be sufficiently severe to result in an ED presentation, hospitalisation or mortality. Acute harms represented in the model were reported as a mean per 100,000 population and include: unintentional injuries (e.g. drowning, falls, fires), alcohol poisoning, road traffic accidents, violence/assault (as a victim or perpetrator) and acute exacerbations resulting from chronic alcohol use disorder (e.g. pancreatitis, gastritis, bleeding and ulcers, mental/behavioural disturbances).

*Chronic harms* Each individual in the model held a risk of developing alcohol-related chronic disease based on their age and average level of consumption (averaged over a 20-year period and applied as a moving window as each individual aged in the model). The duration of a chronic illness from onset to recovery (or death) was calculated for each chronic condition using data from the burden of disease and injury study in Australia (Begg et al. 2007). An annual probability of presenting to ED and/or being hospitalised was included for individuals in the model who developed alcohol-related chronic conditions. The alcohol-related chronic harms represented in the model are lip, oral and pharyngeal cancer, oesophageal cancer, liver cancer, female breast cancer, colorectal cancer, hypertensive diseases, ischaemic heart disease, haemorrhagic stroke, alcoholic liver cirrhosis and alcohol use disorder. Attributable fractions were calculated and applied in the model to ensure that the disease incidences forecasted were limited to those attributable to alcohol consumption. The attributable fraction was derived from the following equation:

$$ {\text{AF}} = \frac{{{\text{RR}} - 1}}{\text{RR}}. $$

The attributable fraction for alcoholic liver cirrhosis is 1. Alcohol-related chronic harms, ED presentations, hospitalisations and mortality generated by the model were reported as a mean per 100,000 population (18 years and over) and plotted over time. For each acute and chronic harm generated, an individual’s ID number, age, sex, alcohol consumption category and type of harm were recorded, creating a synthetic longitudinal dataset of which questions can be asked and traditional statistical analyses conducted.

*Mortality* occurs in the model as a result of alcohol-related acute and chronic harms. In addition, background mortality rates unrelated to alcohol consumption occurred in the model based on age-related mortality curves for NSW (Australian Bureau of Statistics 2014). Upon death of an individual, they were removed from the model.

### Data sources, time frame and calibration

Model assumptions and supporting citations are provided in Online Resource 1—Box 5. The structure and parameterisation of the model drew on a range of evidence and data sources, including systematic reviews (and meta-analyses), longitudinal studies, well-accepted formulas and conceptual models, local survey data and economic data (Online Resource 3). Model structure and parameterisation was reviewed and modified in response to the combined expert knowledge of participating stakeholders through a series of model co-design workshops to reach consensus (Atkinson et al. 2017). In addition, demographic data were sourced from the Australian Bureau of Statistics; drinking behaviour data sourced from the National Drug Strategy Household Survey (NSW and Australian data); and incidence of harm, ED presentations and hospitalisations data were sourced from the Australian Burden of Disease Study dataset and NSW Health administrative datasets. Parameter values, their sources and data used for model calibration are provided in Online Resource 2. The model was calibrated to approximate population statistics from 2011 to 2016 (for alcohol-related harm outputs of the model to be compared against real-world data of that period for validation) and then progresses from January 1, 2017 to December 31, 2021 when simulating interventions. Details regarding model calibration are provided in Online Resource 1—Box 6. The model broadly reproduced historic data patterns across a range of outcome indicators selected on advice from the expert stakeholder group Online Resource 1—Box 7. During the development process, model outputs were iteratively compared to real-world data to ensure that statistics were emerging not as artefacts but due to plausible causal mechanisms.

### Simulation experiments

The baseline scenario (business as usual) used conditions in place across the majority of NSW as of 2016 (i.e. bottle shop closing time of 10 p.m., and licensed venue closing time of 5 a.m.). While the model can simulate a large range of possible combinations of interventions, the following intervention scenarios targeting the reduction of acute alcohol-related harms over the short term were selected as a case study to highlight the policy value of the tool:

*Scenario 1*: 3 a.m. closing time of licensed venues + 1 a.m. ‘lockouts’ rolled out across NSW in 2017.

*Scenario 2*: 3 a.m. closing time of licensed venues rolled out across NSW in 2017.

*Scenario 3*: Expansion of treatment services (to achieve 20% coverage of heavy drinkers) introduced in 2017.

*Scenario 4*: 3 a.m. closing time of licensed venues + 1 a.m. ‘lockouts’ rolled out across NSW + expansion of treatment services (20% coverage of heavy drinkers) introduced in 2017.

### Model outputs

Key outcome indicators against which the impacts of scenarios were compared to the baseline were: (1) incidence of acute alcohol-related harms, (2) ED presentations and (3) hospitalisations. To account for stochasticity, each simulation was run 12 times. A description of the output data processing method is presented in Online Resource 4. Baseline summary statistics for key outcome indicators are presented as a monthly mean per 100,000 population across the 12 runs. In addition, comparison of simulation results between baseline and intervention scenarios was expressed as a percent difference in the mean of two independent samples from the same population.

## Results

**Fig. 3:**
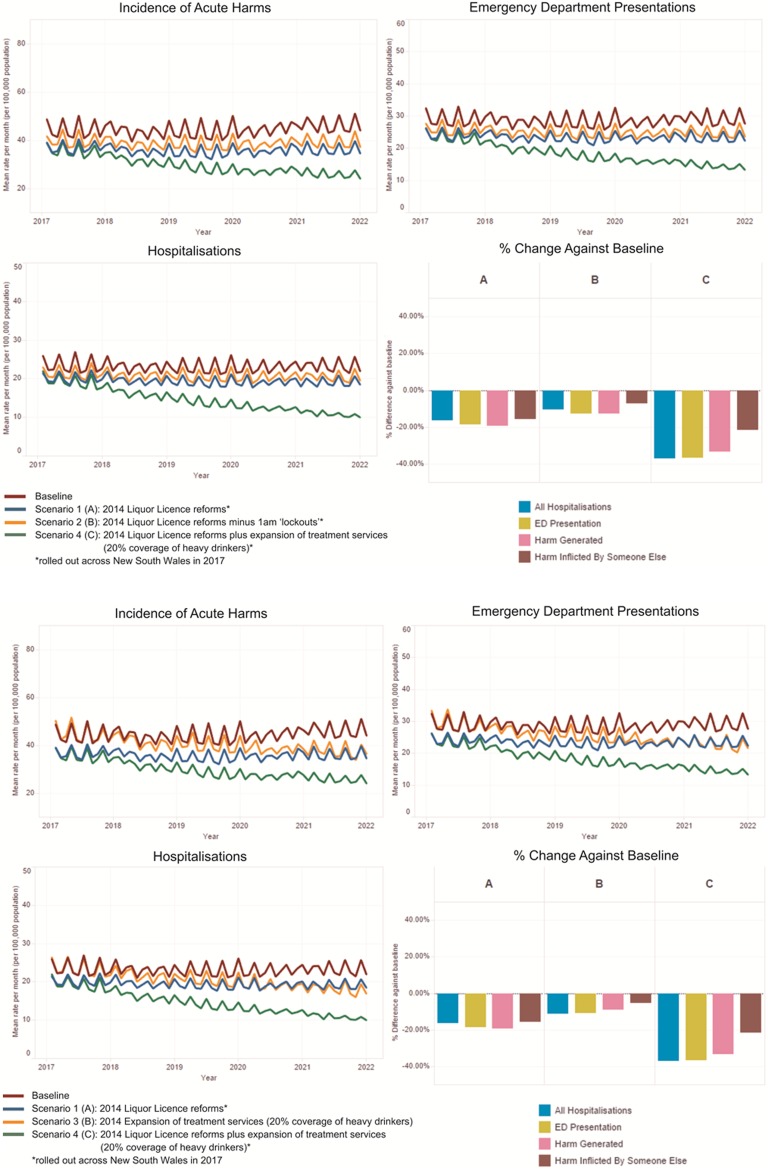
Comparative impacts of scenarios (NSW, Australia; simulated from 2017 to 2021)

The baseline (business as usual) case simulated alcohol-related harms across a 5-year period from January 2017 to December 2021. This baseline case assumed no changes to existing programmes and services. Acute harms contributed 86.7% and chronic harms 13.3% of the total harms generated over the period. Of the mean monthly incidence of acute alcohol-related harms, approximately two-thirds (64.5%) resulted in ED presentation and half were admitted to hospital (52.1%). Tables 1 and 2 provide the summary statistics of the baseline simulation and the outcomes of the simulated scenarios against the baseline. The simulated intervention scenarios were primarily focussed on reducing acute alcohol-related harms; hence, the reporting of model outputs is limited to these impacts. Figure 3 shows graphically the comparative impact of the simulated scenarios against the baseline (business as usual).

**Table 1:** Summary statistics for key outcomes generated from 12 runs of the baseline (NSW, Australian; simulated from 2017 to 2021)

Key outcomes	Mean monthly harms generated (per 100,000 population)	SD	SD % of mean	Margin of error
Incidence of acute harms				
All	44.5	1.8	3.9	± 1.1
Emergency department presentations	28.7	1.0	3.4	± 0.6
Hospitalisations	23.2	0.3	2.8	± 0.2

All results are calculated for a 95% confidence interval

Values are based on a simulated population of approximately 3·6 million

**Table 2:** Summary of reductions from the baseline for each scenario (NSW, Australia; simulated from 2017 to 2021)

	Incidence of acute harms % reduction	Margin of error %	Emergency department presentation % reduction	Margin of error %	Hospitalisations % reduction	Margin of error %
Scenario 1	19.5	± 2.9	18.5	± 2.5	15.7	± 2.1
Scenario 2	12.3	± 2.4	11.9	± 2.1	10.6	± 1.8
Scenario 3	9.0	± 2.9	10.8	± 2.6	12.8	± 2.3
Scenario 4	33.3	± 2.7	36.6	± 2.7	37.2	± 2.6

All results are calculated for a 95% confidence interval

Values are based on a simulated population of approximately 3.6 million

*Scenario 1* estimated a 19.5 ± 2.9% reduction in acute alcohol-related harms, an 18.5 ± 2.5% reduction in ED presentations and a 15.7 ± 2.1% reduction in hospitalisations between 2017 and the end of 2021.

*Scenario 2* estimated a 12.3 ± 2.4% reduction in acute alcohol-related harms, an 11.9 ± 2.1% reduction in ED presentations and a 10.6 ± 1.8% reduction in hospitalisations between 2017 and the end of 2021.

*Scenario 3* estimated a 9 ± 2.9% reduction in acute alcohol-related harms, a 10.8 ± 2.6% reduction in ED presentations and a 12.8 ± 2·3% reduction in hospitalisations between 2017 and the end of 2021.

*Scenario 4* estimated a 33.3 ± 2.7% reduction in acute alcohol-related harms, a 36.6 ± 2.7% reduction in ED presentations and a 37.2 ± 2.6% reduction in hospitalisations between 2017 and the end of 2021.

## Discussion

In Australia, political considerations, community and public health advocacy, industry lobbying and a pro-drinking culture is contributing to a hotly contested debate about the most appropriate course of action to reduce alcohol-related harms (Atkinson et al. 2017; Howard et al. 2014). In addition, Commonwealth, state and territory governments have jurisdiction over different policy areas, resulting in a lack of coherent and coordinated responses to this complex problem (Howard et al. 2014). This work demonstrates the feasibility of participatory development of a contextually relevant agent-based model of alcohol-related harms and highlights its utility in identifying potential policy responses that best leverage limited resources: responses that would otherwise take many years to test and evaluate.

Complex problems such as alcohol misuse and related harms often require multi-strategic cross-agency responses. Commonly, interventions under the jurisdiction of a responding policy agency that are deemed likely to be effective are often packaged, refined based on stakeholder consultation and implemented without adequate understanding of their likely combined effect. In contrast, computer models allow the testing of alternative combinations of interventions and quantify the trade-offs between different combinations of interventions, providing a robust basis on which to negotiate effective and acceptable responses and help avoid a costly trial and error approach. The findings of this study show that scenario 1 (the 3 a.m. closing time of licensed venues + 1 a.m. ‘lockouts’) combined with scenario 3 (the expansion of treatment services to achieve 20% coverage of heavy drinkers) resulted in impacts that are greater than the sum of the scenarios simulated individually, indicating a synergistic effect. This is consistent with the non-additive effects noted as an important corollary of interventions in complex systems (Forrester 1961; Marshall et al. 2015; Rockhill et al. 1998). The potential synergistic effects of interventions delivered by different agencies (health, police, justice and welfare departments and other government, academic and community organisations) may assist in making a compelling case for cross-agency cooperation to deliver coordinated and effective responses to address alcohol misuse and related harms. While the work presented is a case study of the application of participatory agent-based modelling to inform policy and planning in New South Wales (NSW), Australia, the approach is applicable nationally and globally.

*Model limitations and strengths* The baseline model underestimates alcohol poisoning among young adults, but reasonably reproduces this poisoning in the adult population generally. This shortcoming skews the proportion of harms generated by problem groups (i.e. males, individuals under 40 years and individuals with alcohol use disorders), but will be refined in future versions. In addition, while the model initiates new alcohol consumption legal age individuals as time passes, it does not incorporate immigration/migration to or from NSW. This results in a total population reduction over time (~ 0.45%/year). Immigration/migration was not included for reasons of simplicity and lack of data, particularly around drinking category estimates of individuals moving to NSW. However, model outputs are reported as a mean per hundred thousand population, to account for variation in population size over time. In addition, the model does not include domestic violence; hence, total acute harms will be underestimated in both the baseline and simulated runs of the model. Finally, population-level evidence was often used to parameterise individual-level transitions in the agent-based model, due to a lack of more detailed individual-level data regarding the impact of interacting exposures on drinking behaviours. Further collection of individual behavioural trajectory data (using sensor-enabled wearable devices and mobile technologies) would make a valuable contribution to improving model robustness, particularly to improve model representation of the interactions between workplace drinking culture, gender-related social expectations around drinking in particular contexts and peer pressure related to an individual’s social network. However, the primary purpose of the model was to provide decision support capability by estimating the overall comparative impacts of different policy scenarios or combinations of interventions over time against the baseline, rather than providing highly precise predictions of outcome indicators. A strength of the study, and a key innovation in the application of agent-based modelling to complex public health problems, is the explicit engagement of diverse stakeholders in the design and parameterisation of the tool (Atkinson et al. 2015, 2017; Freebairn et al. 2017; O’Donnell et al. 2017). The participatory approach assisted in transparent negotiation and consensus building around the most acceptable policy options in the context of previous, and sometimes contentious, empirical evidence. This approach has developed an effective and acceptable cross-sectoral policy analysis tool to inform responses to reducing alcohol-related harms.

*Ongoing utility as a decision support asset* The NSW alcohol model can be used in an ongoing way as a decision support asset in NSW and may be customised for use in other jurisdictions within Australia or internationally. Models such as this act as a logically consistent framework for integrating disparate data and evidence sources to better understand and address complex problems. In addition, models can be iteratively updated to maintain their utility as a decision support asset and can be used to identify research priorities that will contribute to improving and refining the model and enhancing its value over time. It also allows policy makers to leverage further investment in research, big data collection and analysis, and evaluation of policies and programs. As new evidence comes to light and as new interventions are tested and evaluated, the results can be integrated into the model to help derive more quickly actionable policy and practice recommendations.

## Electronic supplementary material

Below is the link to the electronic supplementary material.

Supplementary material 1 (PDF 666 kb)

Supplementary material 2 (PDF 497 kb)

Supplementary material 3 (PDF 227 kb)

Supplementary material 4 (PDF 433 kb)
