# Supplementary material for: Harnessing advances in computer simulation to inform policy and planning to reduce alcohol-related harms
Source: Int J Public Health. 2017 Oct 19;63(4):537–46. doi: 10.1007/s00038-017-1041-y (PMC5938302; doi:10.1007/s00038-017-1041-y)
Supplement: Supplementary file 1 — Supplementary material 1 (PDF 666 kb) [file 38_2017_1041_MOESM1_ESM.pdf]

Title: ***Harnessing advances in computer simulation to inform policy and planning to reduce alcohol-related harms***

Journal: ***International Journal of Public Health***

Authors: Jo-An Atkinson[a,b,c,d], Dylan Knowles, John Wiggers, Michael Livingston, Robin Room, Ante Prodan, Geoff McDonnell, Eloise O'Donnell, Sandra Jones, Paul S Haber, David Muscatello, Nadine Ezard, Nghi Phung, Louise Freebairn, Devon Indig, Lucie Rychetnik, Jaithri Ananthapavan, and Sonia Wutzke, on behalf of the alcohol modelling consortium.

Affiliations of corresponding author:

[a] Director, Decision Analytics, Sax Institute, Sydney, Australia

[b] The Australian Prevention Partnership Centre, Sax Institute, Sydney, Australia

[c] Sydney Medical School, University of Sydney, Australia

[d] Corresponding author: Decision Analytics, Sax Institute, PO Box K617, Haymarket, NSW, 1240, Australia, Jo-An.Atkinson@saxinstitute.org.au; Ph: +61 2 9188 9537; Fax: +61 2 9188 9501

Online Resource 1: Boxes

Box 1: Harms prioritised for inclusion in the model by the multi-disciplinary stakeholder group

- **Cancers** - lip, oral and pharyngeal cancer, oesophageal cancer, liver cancer, female breast cancer, colorectal cancer
- **Hypertensive diseases**
- **Ischaemic heart disease**
- **Haemorrhagic stroke**
- **Alcoholic liver cirrhosis**
- **Acute exacerbations / presentations** arising from chronic alcohol use disorder
- **Violence (non-domestic)** – harms to self and others
- **Alcohol poisoning**
- **Road traffic accidents** – harms to self and others
- **Accidental injuries** –fire, falls, drowning

Box 2: Interventions prioritised for inclusion in the model by the multi-disciplinary stakeholder group

- **Brief interventions** delivered by health professionals through phone counselling and online services and targeted to moderate alcohol consumers
- **Expansion of alcohol treatment services** (excluding brief interventions) targeted to heavy drinkers
- **Restriction of hours of sale** of alcohol (changing opening hours and bottle shops and licensed venues)
- **'Lockouts'** (preventing admission to venues after a specified time)
- **Restriction of density of licensed venues**
- **Alcohol pricing** (modifications can be made to the average price of standard and low cost alcohol, allowing testing of minimum pricing policies)
- **Advertising restrictions**
- **Enhanced enforcement of liquor laws** i.e. responsible service of alcohol (RSA)

Box 3: Rules that govern the likelihood that an individual will consume alcohol (NSW, Australia) grouped using the COM-B framework

Rules governing 'Capacity' to consume alcohol:

Engaging in alcohol consumption activity is only possible in the model if the individual is:

1. **Awake.** A person cannot consume alcohol if asleep.
2. **Conscious.** Heavily intoxicated / unconscious individuals cannot consume alcohol.
3. **Not Abstinent.** Abstinent people do not partake in alcohol consumption.

Rules governing 'Opportunity' to consume alcohol:

The attractiveness of a particular activity is determined using an Opportunity calculation in the COM-B framework. This calculation represents the probability of engaging in alcohol consumption activities in particular locations based on the following items:

1. **Regulatory factors**. Interventions that make venues less likely to serve an individual (e.g., stringent RSA) make those venues less attractive.
2. **Demographics**. Youth are more drawn to weekend alcohol consumption than weekday consumption.
3. **Access**. If a bottle shop or licensed venue is nearing closing or lockout time, its attractiveness as a destination diminishes.
4. **Density**. Heavy drinkers are drawn to venue-dense precincts.
5. **Price**. As the price of alcohol at licensed venues increases relative to bottle shops, peer events and consuming alcohol at home becomes more desirable. As the relative price decreases, licensed venues become more attractive.

Rules governing 'Motivation' to consume alcohol:

Once at an alcohol consumption venue / event, the amount and rate at which individuals consume alcohol is determined by a Motivation calculation, which is re-calculated hourly and after each drink to account for new contextual constraints or pressures (e.g., out of money, new peers arrive):

1. **Routine**. An individual's past behaviour in a context sets the baseline for their future behaviour. The heavier the consumption category, the higher the baseline. Youth are less affected by prior behaviour than other adults.
2. **Life Phase**. Individuals in their prime working years (30 to 65) consume alcohol more heavily at home. Heavier drinkers will consume alcohol more at home than low drinkers.
3. **Self-Regulation**. Individual self-regulation and availability of funds influence when an individual will cease alcohol consumption. Individuals curb their alcohol consumption when they surpass a threshold defined by their alcohol consumption category and context: heavier and younger drinkers have a higher threshold, and all drinkers temporarily increase this threshold at alcohol-centred events (peer events, precinct outings). Heavier and younger drinkers dedicate more funds to alcohol. Drinkers slowly but non-linearly increase their funds to adjust to price increases, with heavier drinkers having a greater tolerance for extreme price increase than lighter drinkers.
4. **Peer Pressure.** If an individual is with co-workers whose work culture encourages alcohol consumption, or if the individual is a heavier drinker and with friends, the individual feels obliged to consume a minimum

number of alcoholic beverages as a cultural norm. Individuals are also immediately influenced by peer pressure: if the circle of peers consume alcohol heavily on a particular occasion, then the individual will attempt to match that level. Younger drinkers are more heavily influenced by peer pressure.

5. **Intervention Effects**. Interventions that reduce the effect of routine activities (e.g., drug-based treatments for heavy drinkers to reduce ongoing urges to consume alcohol), are applied where appropriate throughout the calculation.

Friend and work networks are gradually changed so they turn over every seven years,(Mollenhorst et al. 2014) and as time progresses funds for alcohol consumption replenish, and alcohol consumption classification changes if required. An individual’s alcohol consumption classification (Abstinent, Low, Moderate, Heavy) updates as their consumption patterns change, which results in corresponding changes to COM-B calculations (e.g., a Moderate drinker is more influenced by past behaviour than a Low drinker).

Box 4: Visualisation of COM-B framework as implemented in the model to determine individual drinking behaviours in NSW, Australia.

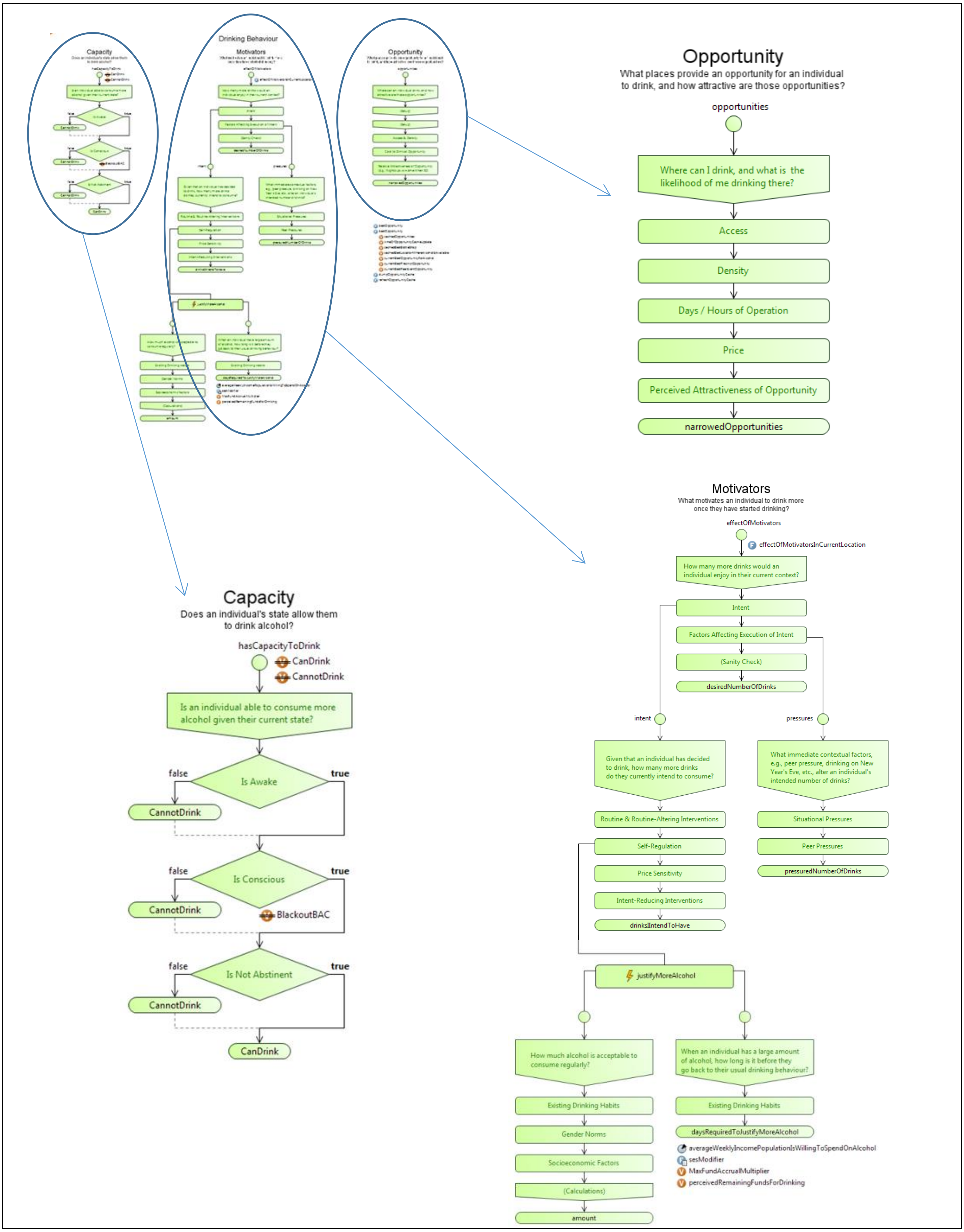

Box 5: Model assumptions and supporting citations

Model Assumptions: Various assumptions about individual alcohol consumption behaviour are made to simplify the model while allowing it to approximate real-world behaviour over time:

1. The number of youth entering the legal drinking population with heavy or moderate alcohol consumption habits is decreasing over time.[1]
2. The heavier an individual consumes alcohol, the more their future activities are shaped by their prior drinking habits[2,3] (e.g., a heavy drinker is likely to consume several alcoholic beverages at home tomorrow if they usually consume several alcoholic beverages at home).
3. Generally, individuals have more income to spend on alcohol during their working years (approximately 30 – 65),[4] and are more inclined to consume alcohol at home during this period of life.[5,6]
4. Generally, as individuals age they become better at regulating their alcohol intake.[7] As individuals increase their alcohol consumption classification (e.g. move from being a moderate drinker to a heavy drinker), this ability for self-regulation of alcohol intake wanes and consumption is tempered more by financial constraints.
5. Alcohol pricing influences the relative attractiveness of location options for alcohol consumption. Heavier drinkers spend more on alcohol and more frequently, and, as alcohol prices increase, will expand their budget more than lighter drinkers. Older drinkers reduce their budget on alcohol.[8]
6. In social situations, individuals are pressured by their peer group to consume alcohol.[7] Peer pressure is strongest in youth and weakest in older adults.[9] Heavier drinkers and individuals assigned to employment groups with heavier alcohol consumption norms feel pressured to have a minimum number of drinks in social situations.[10]
7. Peer events are favoured over alcohol consumption precincts when a friend in an individual's social network is hosting the event.[5,6]
8. Heavy drinkers are attracted to venue-dense precincts.[11]

Underlying these assumptions are simple mathematical relationships designed to capture the concept or intuition they represent. For instance, the influence of peer pressure is assumed to follow an S-curve whose coefficients cause peer pressure to be disproportionately high for youth, stable for the middle years of life, and decline for the elderly; the amount individuals are willing to spend on alcohol expands non-linearly as alcohol price increases and eventually asymptotes to represent hard budgetary limits.

References:

1. NSW Ministry of Health. Trends in alcohol use and health-related harms in NSW: Report of the Chief Health Officer 2016. Sydney: NSW Ministry of Health;http://www.health.nsw.gov.au/hsnsw/Pages/chief-health-officers-report-2016.aspx
2. Stacy AW, Leigh BC, Weingardt KR. Memory accessibility and association of alcohol use and its positive outcomes. Experimental and Clinical Psychopharmacology 1994;2(3):269-282. http://dx.doi.org/210.1037/1064-1297.1032.1033.1269.
3. Townshend JM, Duka T. Attentional bias associated with alcohol cues: differences between heavy and occasional social drinkers. Psychopharmacology (Berl). 2001;157(1):67-74.
4. Australian Bureau of Statistics. Household Expenditure Survey, Australia: Summary of Results, 2009-10. 2011: http://www.abs.gov.au/ausstats/abs@.nsf/Latestproducts/6530.0Media%20Release12009-10?opendocument&tabname=Summary&prodno=6530.0&issue=2009-10&num=&view=
5. Australian Institute of Health and Welfare. 2004 National Drug Strategy Household Survey: Detailed findings. AIHW cat. no. PHE 66. Canberra: Australian Institute of Health and Welfare (Drug Statistics Series No.16);2005.
6. Australian Institute of Health and Welfare. 2010 National Drug Strategy Household Survey report. Drug statistics series no. 25. Cat. no. PHE 145. Canberra: Australian Institute of Health and Welfare;2011.
7. Kuntsche E, Rehm J, Gmel G. Characteristics of binge drinkers in Europe. Soc Sci Med. 2004;59(1):113-127.
8. Marsden Jacob Associates. Bingeing, collateral damage and the benefits and costs of taxing alcohol rationally. Prepared for the Foundation for Alcohol Research and Education;2012: http://fare.org.au/wp-content/uploads/research/FINAL-MJA-Report-Bingeing-Collateral-Damage-and-Taxation-2012.pdf.
9. Steinberg L, Monahan KC. Age differences in resistance to peer influence. Dev Psychol. 2007;43(6):1531-1543.
10. Berry JG, Pidd K, Roche AM, Harrison JE. Prevalence and patterns of alcohol use in the Australian workforce: findings from the 2001 National Drug Strategy Household Survey. Addiction. 2007;102(9):1399-1410.
11. Gruenewald PJ. Regulating availability: how access to alcohol affects drinking and problems in youth and adults. Alcohol Res Health. 2011;34(2):248-256.

Box 6: Details regarding model calibration

The model calibration process presented a number of challenges. The model had significant runtime and many output indicators that were required to be reproduced to generate confidence in model forecasts (e.g. the drinking patterns of males and females by age groups, a broad range of alcohol related harms, percent alcohol consumed among the top 5%, 10%, 50% of drinkers etc.). In addition, parameters affected multiple output indicators, making their calibration across numerous data sets important but highly labour intensive, as standard automated exhaustive search calibration methods were ill-suited due to combinatorial explosion. Targets to calibrate towards were also inherently fuzzy, with multiple data sources providing similar but not equivalent values for a given outcome indicator. For example, the percent of young adults drinking at lifetime risk or single-occasion risk levels varied between data sources. In most cases, an approximate margin of error of 15-20% was considered acceptable. By maximizing the number of outputs achieving this low percent error, it was observed that calibration produced reliable outputs in areas that were not explicitly calibrated towards. An additional useful strategy was to group calibration metrics and achieve calibration incrementally. Drinking behaviour indicators were the foundation of the model and consequently received the majority of focus and time. This was followed by an overall acute and chronic harm indicator group, then certain specific context-based or severity of drinking episode based harms (e.g. Alcohol poisoning and road traffic accidents), and finally health system burden metrics (ED presentations and hospitalisations). This hierarchy of calibration approach made the calibration challenges surmountable.

Box 7: Outcome indicators the model reproduces

- Percent of 18-24-year-olds consuming alcohol at lifetime risk levels, by sex
- Percent of entire population consuming alcohol at lifetime risk levels, by sex and age
- Proportion exceeding single occasion risk guidelines at least monthly, by age
- Average number of drinks per day, by alcohol consumption classification
- Alcohol attributable harms per 100,000 population by sex and harm type (acute / chronic)
- Emergency Department presentations by sex
- Percent of alcohol consumed by the top 5%, 10%, and 50% of drinkers
- Percent of alcohol consumed from bottle shops and licensed venues
- Percent of people who are abstinent, consume alcohol less than weekly, consume alcohol weekly, and consume alcohol daily
- Percent of people in Heavy, Moderate, Low, and Abstinent alcohol consumption categories
- Distribution of select harm types, by age and sex (e.g., violence)
- Outcomes seen in known interventions, e.g., decrease in violence, drinks per day, etc., following implementation.
