# Supplementary material for: Harnessing advances in computer simulation to inform policy and planning to reduce alcohol-related harms
Source: Int J Public Health. 2017 Oct 19;63(4):537–46. doi: 10.1007/s00038-017-1041-y (PMC5938302; doi:10.1007/s00038-017-1041-y)
Supplement: Supplementary file 2 — Supplementary material 2 (PDF 497 kb) [file 38_2017_1041_MOESM2_ESM.pdf]

Title: ***Harnessing advances in computer simulation to inform policy and planning to reduce alcohol-related harms***

Journal: ***International Journal of Public Health***

Authors: Jo-An Atkinson[a,b,c,d], Dylan Knowles, John Wiggers, Michael Livingston, Robin Room, Ante Prodan, Geoff McDonnell, Eloise O'Donnell, Sandra Jones, Paul S Haber, David Muscatello, Nadine Ezard, Nghi Phung, Louise Freebairn, Devon Indig, Lucie Rychetnik, Jaithri Ananthapavan, and Sonia Wutzke, on behalf of the alcohol modelling consortium.

Affiliations of corresponding author:

[a] Director, Decision Analytics, Sax Institute, Sydney, Australia

[b] The Australian Prevention Partnership Centre, Sax Institute, Sydney, Australia

[c] Sydney Medical School, University of Sydney, Australia

[d] Corresponding author: Decision Analytics, Sax Institute, PO Box K617, Haymarket, NSW, 1240, Australia, Jo-An.Atkinson@saxinstitute.org.au; Ph: +61 2 9188 9537; Fax: +61 2 9188 9501

Online Resource 2: Parameter estimates and data sources (NSW alcohol model)

Variable	Description	Value	Source	Comments
**P(Youth Group Change Venue Per Hour)**	The probability that a group of young people will change venues in a drinking precinct per hour.	0.12	Scott, N et al. SimDrink: An agent-based netlogo model of young, heavy drinkers for conducting alcohol policy experiments. JASSS, 2016.	
**P(Host Event)**	The probability that someone will host a private event on any given evening. This is a base rate that does not take into consideration alcohol prices.	0.033 (i.e. approximately once a month)	Unconfirmed estimate	
**Alcohol Metabolism Per Hour (ml / hour)**	The amount of alcohol a person metabolizes per hour, e.g., 15ml per hour.	15 ml	Widmark EMP. Widmark Equation for predicting Blood Alcohol Concentration: http://www.mayomedicallaboratories.com/test-info/drug-book/alcohol.html. 1981.	
**Average Weekly Income Population Is Willing To Spend On Alcohol**	The amount of money the average person is willing to spend on alcohol from a bottle shop each week.	Value = $25	Approximated from:<br>Australian Bureau of Statistics (2011). Household Expenditure Survey, Summary of Results, 2009-10, Cat. No. 6530.0 Canberra, Australian Bureau of Statistics.	
**Default Social Network Size**	The average size of an individual's friend and co-worker social networks.	Initial value = 8	Derived from http://www.gallup.com/poll/112723/Gallup-Daily-US-Consumer-Spending.aspx	
**Widmark equation**	Values for the Widmark equation used to compute BAC	Distribution ratio:<br>Male = 0.68<br>Female = 0.55<br>Ethanol Density (g/ml) = 0.8	Widmark Equation for predicting Blood Alcohol Concentration: http://www.mayomedicallaboratories.com/test-info/drug-book/alcohol.html. 1981.	

**Standard drink**	Information about the contents of a standard drink of alcohol e.g. ml alcohol per unit.	Alcohol (g) = 10<br>Alcohol (ml) = 12.5	NHMRC Australian Guidelines to reduce health risks from drinking alcohol. Commonwealth of Australia: National Health and Medical Research Council; 2009 Jan 1: http://www.nhmrc.gov.au/_files_nhmrc/publications/attachments/ds10-alcohol.pdf]
**Generic Drink Price**	The average price people pay for one generic drink in various contexts.	At Bar = $6<br>At Bottle Shop:<br>Low quality = $1.00<br>Normal quality = $1.80	Figures approximated from:<br>Callinan, S. et al. (2015) Who Purchases Low-Cost Alcohol? Alcohol and Alcoholism. 50(6).
**Single Occasion Risk Drinking**	The number of standard drinks required (inclusive) for a drinking episode to be considered risky drinking.	4	NHMRC Australian Guidelines to reduce health risks from drinking alcohol. Commonwealth of Australia: National Health and Medical Research Council; 2009 Jan 1: http://www.nhmrc.gov.au/_files_nhmrc/publications/attachments/ds10-alcohol.pdf]
**Lifetime risk drinking**	The number of standard drinks per day beyond which an individual is said to be a lifetime risk of chronic alcohol-related harms.	2	NHMRC Australian Guidelines to reduce health risks from drinking alcohol. Commonwealth of Australia: National Health and Medical Research Council; 2009 Jan 1: http://www.nhmrc.gov.au/_files_nhmrc/publications/attachments/ds10-alcohol.pdf]
**Average Commute**	The average time people need to commute to work.	0.75 (45 minutes)	Estimate
**Average Preloading Session**	The average time spent preloading before going to a licensed venue.	1 hour	Estimate

**Average Time Spent Getting To Peer Event**	The average time people need to get to and from social drinking events, like parties or things at friends' homes.	0.25 (15 minutes)	Estimate	
**Average Time Spent At Bottle Shop**	The time people spend at the bottle shop looking for liquor.	0.17 (approximately 10 mins)	Estimate	
**Physical Drinks Acquired Per Bottle Shop Trip**	The average number of physical drinks a person retrieves when they go to a bottle shop.	6	Estimate	
**Blackout BAC**	The BAC level at which someone blacks out and is therefore unable to drink more.	0.40	Estimate	
**Time to Drink (Minutes)**	The time required to drink one physical serving of a given type of alcohol.	15 minutes	Estimate	
**Price Sensitivity parameters:**				
**Time to Adjust to New Price (Months)**	The number of months required to adjust to a sudden price increase.	1 month	Estimate	
**Moderate Drinker Price Sensitivity***	Price elasticities for moderate drinkers.*	-0.4971*	Marsden Jacob Associates. Bingeing, collateral damage and the benefits and costs of taxing alcohol rationally. Report to the Foundation for Alcohol Research and Education, October 2012: http://bettertax.gov.au/files/2015/06/Foundation_for_Alcohol_Research_and_Education_Submission_2.pdf	Beer data is used as a simplifying assumption<br>*NB These categories are used for price elasticity classifications ONLY. Please see details in rows below.
**Hazardous Drinker Price Sensitivity***	Price elasticities for hazardous drinkers.*	-0.3926*	Marsden Jacob Associates. Bingeing, collateral damage and the benefits and costs of taxing alcohol	Beer data is used as a simplifying assumption<br>*NB These categories are

			rationally. Report to the Foundation for Alcohol Research and Education, October 2012: http://bettertax.gov.au/files/2015/06/Foundation_for_Alcohol_Research_and_Education_Submission_2.pdf	used for price elasticity classifications ONLY. Please see details in rows below.
**Harmful Drinker Price Sensitivity***	Price elasticities for harmful drinkers.*	-0.2628*	Marsden Jacob Associates. Bingeing, collateral damage and the benefits and costs of taxing alcohol rationally. Report to the Foundation for Alcohol Research and Education, October 2012: http://bettertax.gov.au/files/2015/06/Foundation_for_Alcohol_Research_and_Education_Submission_2.pdf	Beer data is used as a simplifying assumption<br>*NB These categories are used for price elasticity classifications ONLY. Please see details in rows below.
**Moderate drinker category**	Maximum drinks	Standard drinks per day = 2; drinks per week = 14.	Marsden Jacob Associates. Bingeing, collateral damage and the benefits and costs of taxing alcohol rationally. Report to the Foundation for Alcohol Research and Education, October 2012: http://bettertax.gov.au/files/2015/06/Foundation_for_Alcohol_Research_and_Education_Submission_2.pdf	
**Hazardous drinker category**	Maximum drinks	Standard drinks per week male = 40, female = 28.	Marsden Jacob Associates. Bingeing, collateral damage and the benefits and costs of taxing alcohol rationally. Report to the Foundation for Alcohol Research and Education, October 2012: http://bettertax.gov.a	

			u/files/2015/06/Foundation_for_Alcohol_Research_and_Education_Submission_2.pdf	
**Average duration of chronic harm in years**	Duration of a chronic illness from onset to recovery (or death)	Liver cancer = 3.79 years<br>Breast cancer = 4.23 years<br>Colorectal cancer = 3.3 years<br>Mouth and oropharynx cancers = 3.93 years<br>Oesophagus cancer = 3.6 years<br>Hypertensive heart disease = 7.96 years<br>Ischaemic heart disease = 7.86 years<br>Stroke = 9.35 years<br>Liver cirrhosis = 4 years	Calculated for NSW 2010 from the Australian Burden of Disease dataset:<br>Begg S VT, Barker B, Stevenson C, Stanley L & Lopez A. Burden of disease and injury in Australia, 2003. Cat. no. PHE 82 Canberra: Australian Institute of Health and Welfare http://www.aihw.gov.au/publications/index.cfm/title/10317;2007.	Duration given for stroke is for those that survive beyond 28 days. It is assumed from the data that 20% of stroke patients do not survive beyond 28 days.
**Average duration or acute harm in weeks**	Average time it takes to recover from an acute harm/injury	4 weeks	Calculated for NSW 2010 from the Australian Burden of Disease dataset	
**Probability of long term disability**	Probability that an acute harm will result in long term disability	0.087	Calculated for NSW 2010 from the Australian Burden of Disease dataset	
**P(ED Admission)**	The probability that an individual will be admitted to emergency when suffering an acute harm.	0.1	Estimate	
**P(Hospitalization | ED Admission)**	The probability that an individual will be hospitalised if they present to an emergency department for harm.	0.1	Estimate	
**Injuries (falls, fires, drownings)**	Relative risks by average consumption	1 = 1.05<br>2 = 1.11	Corrao et al 2004. A meta-analysis of alcohol consumption	

	of standard drinks per day	3 = 1.16<br>4 = 1.22<br>5 = 1.28<br>6 = 1.35<br>7 = 1.42<br>8 = 1.49<br>9 = 1.55<br>10 = 1.63<br>11 = 1.65<br>12+ = 1.68	and the risk of 15 diseases. Prev Med 38: 613-19	
**Alcohol poisoning (unconsciousness and death)**	Probability of becoming unconscious given a certain blood alcohol concentration.	BAC = 0.3-0.399, p = 0.6;<br>BAC = 0.4-0.499, p = 0.8;<br>BAC = 0.5+, p = 0.9.	Expert estimate	It was estimated that only 60% of people that become unconscious go to ED (and are diagnosed with alcohol poisoning).
**Hospitalisation rate for ED alcohol poisoning**	Hospitalisation rate given ED presentation for alcohol poisoning.	5%	Expert estimate	Assumption that 95% recover overnight in ED.
**Death from alcohol poisoning**	Probability of death from alcohol poisoning given a certain blood alcohol concentration.	BAC = 0.3-0.399,<br>*p* = 0.4;<br>BAC = 0.4-0.499,<br>*p* = 0.6;<br>BAC = 0.5+,<br>*p* = 0.8.	Expert estimate	
**Road traffic accidents (deaths)**	Risk estimates for alcohol exposure and motor vehicle driver and motorcycle rider deaths as a result of the accident.	Refer to table below, from page 34 of adjacent reference.	Lloyd C 1992. Alcohol and fatal road accidents: estimates of risk in Australia 1983. Accident Analysis and Prevention 24:339–48 (as cited by Ridolfo B & Stevenson C (2001) The Quantification of Drug-caused Mortality and Morbidity in Australia, 1998. AIHW cat. no. PHE 29. Drug Statistics<br>Series no. 7. Australian Institute	

		Blood alcohol concentration					
		>0–0.05		>0.05–0.10		>0.10	
Sex	Age	RR	95% CI	RR	95% CI	RR	95% CI
Male	<21	2.01	1.12–3.60	10.38	5.31–20.29	67.35	35.40–128.11
	21–29	1.14	0.58–2.23	4.96	2.59– 9.49	113.88	73.24–177.07
	30–50	1.33	0.66–2.69	5.74	2.92–11.27	142.43	89.15–227.56
	>50	1.44	0.61–3.37	3.75	1.38–10.19	45.15	22.58–90.27
	All ages	1.45	1.04–2.04	5.86	4.18–8.23	96.82	75.03–124.94

		Blood alcohol concentration			
		>0.01–0.10		>0.10	
		RR	95% CI	RR	95% CI
Female	<30	1.78	0.72–4.39	72.59	34.83–151.29
	30+	2.22	0.99–4.96	52.36	18.86–145.37
	All ages	2.01	1.10–3.66	65.17	36.19–117.38

<table>
<tr><td></td><td></td><td></td><td>of Health and Welfare, Canberra).</td></tr>
<tr><td>Road traffic accidents (hospitalisations)</td><td>Risk estimates for hospitalisations for road traffic accidents based on blood alcohol concentration.</td><td>Refer to Table below, from page 36 of adjacent reference.</td><td rowspan="2">Ridolfo B & Stevenson C (2001) The Quantification of Drug-caused Mortality and Morbidity in Australia, 1998. AIHW cat. no. PHE 29. Drug Statistics Series no. 7. Australian Institute of Health and Welfare, Canberra).</td></tr>
<tr><td colspan="3"><table>
<tr><th>Blood alcohol concentration</th><th>RR</th></tr>
<tr><td>0.01–0.03</td><td>0.69</td></tr>
<tr><td>0.04–0.06</td><td>1.83</td></tr>
<tr><td>0.07–0.09</td><td>3.20</td></tr>
<tr><td>0.10 and over</td><td>12.94</td></tr>
</table></td></tr>
<tr><td>Violence</td><td>Relative risk estimates used for violence</td><td>Unable to find relative risk estimates for violence, therefore the general injuries RRs based on number of drinks consumed in the episode were used.</td><td>Ridolfo B & Stevenson C (2001) The Quantification of Drug-caused Mortality and Morbidity in Australia, 1998. AIHW cat. no. PHE 29. Drug Statistics Series no. 7. Australian Institute of Health and Welfare, Canberra.</td></tr>
<tr><td>Lip, oral and pharyngeal cancer</td><td>Relative risks by average standard drink consumption for lip, oral and pharyngeal cancer</td><td>1 = 1.28
2 = 1.65
3 = 2.12
4 = 2.72
5 = 3.32
6 = 4.06
7 = 4.95
8 = 5.58
9 = 6.05
10 = 6.69
11 = 7.77
12+ = 8.85</td><td>Corrao et al 2004. A meta-analysis of alcohol consumption and the risk of 15 diseases. Prev Med 38: 613-19.</td></tr>
<tr><td>Oesophageal cancer</td><td>Relative risks by average standard drink consumption for oesophageal cancer</td><td>1 = 1.14
2 = 1.28
3 = 1.46
4 = 1.65
5 = 1.87
6 = 2.23</td><td>Corrao et al 2004. A meta-analysis of alcohol consumption and the risk of 15 diseases. Prev Med 38: 613-19.</td></tr>
</table>

		7 = 2.46<br>8 = 2.94<br>9 = 3.49<br>10 = 3.67<br>11 = 4.06<br>12+ = 4.95	
**Liver cancer**	Relative risks by average standard drink consumption for liver cancer.	1 = 1.09<br>2 = 1.16<br>3 = 1.25<br>4 = 1.35<br>5 = 1.42<br>6 = 1.49<br>7 = 1.58<br>8 = 1.65<br>9 = 1.79<br>10 = 1.84<br>11 = 1.92<br>12+ = 2.01	Corrao et al 2004. A meta-analysis of alcohol consumption and the risk of 15 diseases. Prev Med 38: 613-19.
**Breast cancer (women)**	Relative risks by average standard drink consumption for breast cancer (women).	1 = 1.11<br>2 = 1.22<br>3 = 1.28<br>4 = 1.48<br>5 = 1.57<br>6 = 1.65<br>7 = 1.82<br>8 = 2.12<br>9 = 2.27<br>10 = 2.46<br>11 = 2.66<br>12+ = 2.94	Corrao et al 2004. A meta-analysis of alcohol consumption and the risk of 15 diseases. Prev Med 38: 613-19.
**Colorectal cancer**	Relative risks by average standard drink consumption for colorectal cancer.	1 = 1.08<br>2 = 1.14<br>3 = 1.23<br>4 = 1.31<br>5 = 1.33<br>6 = 1.39<br>7 = 1.48<br>8 = 1.52<br>9 = 1.57	Corrao et al 2004. A meta-analysis of alcohol consumption and the risk of 15 diseases. Prev Med 38: 613-19.

		10 = 1.63<br>11 = 1.69<br>12+ = 1.71		
**Hypertensive diseases**	Relative risks by average standard drink consumption for hypertensive diseases.	1 = 1.16<br>2 = 1.35<br>3 = 1.57<br>4 = 1.82<br>5 = 2.12<br>6 = 2.4<br>7 = 2.72<br>8 = 3.25<br>9 = 3.67<br>10 = 4.26<br>11 = 4.85<br>12+ = 5.75	Corrao et al 2004. A meta-analysis of alcohol consumption and the risk of 15 diseases. Prev Med 38: 613-19.	
**Ischaemic heart disease**	Relative risks by average standard drink consumption for ischaemic heart disease	1 = 1<br>2 = 1<br>3 = 1<br>4 = 1<br>5 = 1<br>6 = 1<br>7 = 1<br>8 = 1.05<br>9 = 1.11<br>10 = 1.12<br>11 = 1.19<br>12+ = 1.25	Corrao et al 2004. A meta-analysis of alcohol consumption and the risk of 15 diseases. Prev Med 38: 613-19	For the purposes of this model it was assumed that there was no protective effect of drinking at low to moderate levels (for ischemic heart disease) based on Stockwell et al. Do 'moderate drinkers have reduced mortality risk? A systematic review and meta-analysis of alcohol consumption and all-cause mortality. Journal of Studies on alcohol and drugs. 2016; 77(2): 185-198.
**Haemorrhagic stroke**	Relative risks by average standard drink consumption for haemorrhagic stroke.	1 = 1<br>2 = 1.14<br>3 = 1.34<br>4 = 1.62<br>5 = 1.82<br>6 = 2.34<br>7 = 2.72<br>8 = 3.49<br>9 = 4.06<br>10 = 4.95	Corrao et al 2004. A meta-analysis of alcohol consumption and the risk of 15 diseases. Prev Med 38: 613-19.	

		11 = 6.05<br>12+ = 7.39		
**Alcoholic Liver cirrhosis**	Annual probability of liver cirrhosis by standard drink consumption per day.	< 1 = 0.00074<br>1 – 1.99 = 0.00032<br>2 – 3.99 = 0.00074<br>4 – 5.99 = 0.00285<br>6 – 10 = 0.00537<br>10+ = 0.00671<br>NB – all figures rounded to 5 decimal points	Becker et al 1996, Prediction of risk of liver disease by alcohol intake, sex, and age: A prospective population study. Hepatology journal 23(5): 1025	Attributable risk assumed to be 1.
**Hospitalization Rate Per Year Distribution**	The distribution of hospitalizations per year for people living with chronic disease	Cancers	Australian Institute of Health and Welfare & Australasian Association of Cancer Registries 2012. Cancer in Australia: an overview, 2012. Cancer series no. 74. Cat. no. CAN 70. Canberra: AIHW.	
		Alcohol use disorders	Data provided by NSW Ministry of Health	
		Stroke	Australian Institute of Health and Welfare 2013. Stroke and its management in Australia: an update. Cardiovascular disease series no. 37. Cat. no. CVD 61. Canberra: AIHW.	
		Liver cirrhosis	Mortality rate of alcoholic liver disease and risk of hospitalization for alcoholic liver cirrhosis, alcoholic hepatitis and alcoholic liver failure in Australia between 1993 and 2005. Internal Medicine Journal, 2011.	

		Other chronic conditions	Feyer, A. et al (2014) Chronic disease program management: final report Oct 2014. Figure 49, page 119.	Assumed a long tail distribution
**ED Presentation Rate Per Year**	The distribution of emergency department presentations per year for individuals with chronic disease.	Figure 49, page 119	Feyer, A. et al (2014) Chronic disease program management: final report Oct 2014.	Assumed a long tail distribution
**Delinquent Behaviour Multiplier By Age_females**	Describes how much more likely females are to get into delinquent behaviour per litre of total alcohol consumption by age.	Derived from figure 18 in the adjacent citation.	NHMRC Australian Guidelines to reduce health risks from drinking alcohol. Commonwealth of Australia: National Health and Medical Research Council; 2009 Jan 1: http://www.nhmrc.gov.au/_files_nhmrc/publications/attachments/ds10-alcohol.pdf]	Also informed by Figures 11, 12 and 13 in the adjacent citation.
**Delinquent Behaviour Multiplier By Age_males**	Describes how much more likely males are to get into delinquent behaviour given their age and alcohol consumption.	Derived from figure 18 in the adjacent citation.	NHMRC Australian Guidelines to reduce health risks from drinking alcohol. Commonwealth of Australia: National Health and Medical Research Council; 2009 Jan 1: http://www.nhmrc.gov.au/_files_nhmrc/publications/attachments/ds10-alcohol.pdf]<br>Figures 18	Also informed by Figures 11, 12 and 13 in the adjacent citation.
**Hazardous Behaviour Multiplier By Age_females**	Describes how much more likely females are to get into hazardous behaviour per litre of total alcohol consumption by age.	Derived from figure 17 in the adjacent citation.	NHMRC Australian Guidelines to reduce health risks from drinking alcohol. Commonwealth of Australia: National Health and Medical Research Council; 2009 Jan 1: http://www.nhmrc.gov.au/_files_nhmrc/publications/attachments/ds10-alcohol.pdf]	Also informed by Figures 11, 12 and 13 in the adjacent citation.
**Hazardous Behaviour**	Describes how much more likely males are to get into hazardous	Derived from figure 17 in the adjacent citation.	NHMRC Australian Guidelines to reduce health risks from	Also informed by Figures 11, 12 and

**Multiplier By Age_males**	behaviour per litre of total alcohol consumption by age.	drinking alcohol. Commonwealth of Australia: National Health and Medical Research Council; 2009 Jan 1.	13 in the adjacent citation.

Additional data and evidence sources used to inform design of the model

- National Drug Strategy Household Survey data – 2010-2013
- Centre for Epidemiology and Evidence. Trends in alcohol use and health-related harms in NSW: Report of the Chief Health Officer 2016. Sydney: NSW Ministry of Health: http://www.health.nsw.gov.au/hsnsw/Pages/chief-health-officers-report-2016.aspx
- Fulde GW, Smith M, Forster SL. Presentations with alcohol-related serious injury to a major Sydney trauma hospital after 2014 changes to liquor laws. The Medical journal of Australia. Nov 2 2015;203(9):366.
- Couples in Australia, ABS Australian Social Trends. 4102.0. 2009
- Combined Admitted Patient Epidemiology Data, NSW Ministry of Health, Secure Analytics for Population Health Research and Intelligence: http://www.health.nsw.gov.au/epidemiology/Pages/Population-health-data-warehouse.aspx
- Emergency Department Records for Epidemiology, NSW Ministry of Health Secure Analytics for Population Health Research and Intelligence.
- Gao C, Ogeil RP, & Lloyd B (2014). Alcohol's burden of disease in Australia. Canberra: FARE and VicHealth in collaboration with Turning Point.
- Martineau F et al. Population-level interventions to reduce alcohol-related harm: an overview of systematic reviews. Preventive Medicine, 2013; 57: 278-296.
- Francois, L et al. SimARC: An ontology-driven behavioural model of alcohol abuse. The Third International Conference on Advances in System Simulation SimARC, 2011.
- Indig D et al. Why are alcohol-related emergency department presentations under-detected? An exploratory study using nursing triage text. Drug and Alcohol Review, 2008; **27: 584-590**.
- Begg S, Vos T, Barker B, Stevenson C, Stanley L & Lopez A (2007). Burden of disease and injury in Australia, 2003. Cat. no. PHE 82. Canberra: Australian Institute of Health and Welfare http://www.aihw.gov.au/publications/index.cfm/title/10317
- Cobiac L et al. Cost-effectiveness of interventions to prevent alcohol-related disease and injury in Australia. Addiction, 2009; 104: 1646-1655.
- International Center for Alcohol Policies (ICAP). (2009). Determinants of Drinking. ICAP Health (Online). Available at: https://preventionconversation.files.wordpress.com/2015/07/determinants-of-drinking-issues-briefing.pdf
- Marsden Jacob Associates. Bingeing, collateral damage and the benefits and costs of taxing alcohol rationally. Report to the Foundation for Alcohol Research and Education, October 2012: http://bettertax.gov.au/files/2015/06/Foundation_for_Alcohol_Research_and_Education_Submission_2.pdf
- Australian Health Survey: First Results, 2011–12 — New South Wales weights by age and sex, ABS: 43640DO001_20112012: http://www.abs.gov.au/AUSSTATS/abs@.nsf/DetailsPage/4364.0.55.0012011-12?OpenDocument
- Life Tables, States, Territories and Australia, 2011-2013: NSW Data: ABS: 3302.0.55.001 http://www.abs.gov.au/ausstats/abs@.nsf/mf/3302.0.55.001
- Deaths, Australia, 2014: NSW data: ABS: 3302.0 http://www.abs.gov.au/AUSSTATS/abs@.nsf/DetailsPage/3302.02014?OpenDocument

- Marriages and Divorces, Australia, 2014: ABS: 3310.0: http://www.abs.gov.au/ausstats/abs@.nsf/mf/3310.0
- Cancer in New South Wales: Incidence Report 2009. Cancer Institute NSW, NSW Government. 2014. ISBN 978-1-74187-966-7
- Roche AM et al. Evidence review: The social determinants of inequities in alcohol consumption and alcohol-related health outcomes. Victorian Health Promotion Foundation, 2015: http://nceta.flinders.edu.au/files/8014/5758/1219/EN607.pdf
- Australian Bureau of Statistics (2011) 6530.0 - Household Expenditure Survey, Australia: Summary of Results, 2009-10: http://www.abs.gov.au/ausstats/abs@.nsf/Latestproducts/6530.0Media%20Release12009-10?opendocument&tabname=Summary&prodno=6530.0&issue=2009-10&num=&view=
- Stacy AW, Leigh BC, Weingardt KR. Memory accessibility and association of alcohol use and its positive outcomes. Experimental and Clinical Psychopharmacology 1994;2(3):269-282. http://dx.doi.org/210.1037/1064-1297.1032.1033.1269.
- Townshend JM, Duka T. Attentional bias associated with alcohol cues: differences between heavy and occasional social drinkers. Psychopharmacology (Berl). 2001;157(1):67-74.
- Australian Institute of Health and Welfare. 2004 National Drug Strategy Household Survey: Detailed findings. AIHW cat. no. PHE 66. Canberra: Australian Institute of Health and Welfare (Drug Statistics Series No.16);2005.
- Australian Institute of Health and Welfare. 2010 National Drug Strategy Household Survey report. Drug statistics series no. 25. Cat. no. PHE 145. Canberra: Australian Institute of Health and Welfare;2011.
- Kuntsche E, Rehm J, Gmel G. Characteristics of binge drinkers in Europe. Soc Sci Med. 2004;59(1):113-127.
- Steinberg L, Monahan KC. Age differences in resistance to peer influence. Dev Psychol. 2007;43(6):1531-1543.
- Berry JG, Pidd K, Roche AM, Harrison JE. Prevalence and patterns of alcohol use in the Australian workforce: findings from the 2001 National Drug Strategy Household Survey. Addiction. 2007;102(9):1399-1410.
- Gruenewald PJ. Regulating availability: how access to alcohol affects drinking and problems in youth and adults. Alcohol Res Health. 2011;34(2):248-256.
