# Supplementary material for: Harnessing advances in computer simulation to inform policy and planning to reduce alcohol-related harms
Source: Int J Public Health. 2017 Oct 19;63(4):537–46. doi: 10.1007/s00038-017-1041-y (PMC5938302; doi:10.1007/s00038-017-1041-y)
Supplement: Supplementary file 3 — Supplementary material 3 (PDF 227 kb) [file 38_2017_1041_MOESM3_ESM.pdf]

Title: ***Harnessing advances in computer simulation to inform policy and planning to reduce alcohol-related harms***

Journal: ***International Journal of Public Health***

Authors: Jo-An Atkinson[a,b,c,d], Dylan Knowles, John Wiggers, Michael Livingston, Robin Room, Ante Prodan, Geoff McDonnell, Eloise O'Donnell, Sandra Jones, Paul S Haber, David Muscatello, Nadine Ezard, Nghi Phung, Louise Freebairn, Devon Indig, Lucie Rychetnik, Jaithri Ananthapavan, and Sonia Wutzke, on behalf of the alcohol modelling consortium.

Affiliations of corresponding author:

[a] Director, Decision Analytics, Sax Institute, Sydney, Australia

[b] The Australian Prevention Partnership Centre, Sax Institute, Sydney, Australia

[c] Sydney Medical School, University of Sydney, Australia

[d] Corresponding author: Decision Analytics, Sax Institute, PO Box K617, Haymarket, NSW, 1240, Australia, Jo-An.Atkinson@saxinstitute.org.au; Ph: +61 2 9188 9537; Fax: +61 2 9188 9501

Online Resource 1: Summary of data sources and role of expert participants (NSW alcohol model)

Types of content knowledge contributed by multi-disciplinary team	Types of literature and data used
• Alcohol and its mechanisms of effect;<br>• Determinants and mediators of alcohol consumption and misuse;<br>• Individual and public health treatments, interventions, programs and their effect;<br>• Current policy contexts and decision processes;<br>• Availability of, and access to, data sources and evidence;<br>• Strengths and limitations of available data sources;<br>• Prioritisation of mechanisms, factors, treatments.	**National and state data, for example:**<br>Demographic data from the Australian Bureau of Statistics<br>Administrative datasets from Centre for Epidemiology and Evidence. HealthStats NSW. Sydney: NSW Ministry of Health. Available at: www.healthstats.nsw.gov.au. Accessed [July 2015].<br>Burden of disease data from Begg S, Vos T, Barker B, et al. Burden of disease and injury in Australia, 2003. Cat. no. PHE 82 Canberra: Australian Institute of Health and Welfare http://www.aihw.gov.au/publications/index.cfm/title/10317, 2007.<br>**Survey data, for example:**<br>Australian Institute of Health and Welfare. National Drug Strategy Household Survey detailed report: 2013. Canberra. Available at: http://www.aihw.gov.au/publication-detail/?id=60129549469 2014.<br>Tindall J, Groombridge D, Wiggers J, et al. Alcohol-related crime in city entertainment precincts: Public perception and experience of alcohol-related crime and support for strategies to reduce such crime. Drug and alcohol review 2015.<br>**Longitudinal data, for example:**<br>Becker et al 1996, Prediction of risk of liver disease by alcohol intake, sex, and age: A prospective population study. Hepatology Journal 23(5): 1025<br>**Accepted formulas, for example:**<br>Widmark equation for Blood Alcohol Concentration: Widmark, E.M.P., Principles and Applications of Medicolegal Alcohol Determination, Davis, CA: Biomedical Publications, 1981, pp. 107-108.<br>**Well supported theoretical models, for example:**<br>Michie S, van Stralen MM, West R. The behaviour change wheel: a new method for characterising and designing behaviour change interventions. Implementation science : IS. 2011;6:42.<br>**Systematic reviews and meta-analyses, for example:**<br>Corrao et al 2004. A meta-analysis of alcohol consumption and the risk of 15 diseases. Prev Med 38: 613-19<br>Martineau et al. (2013). Population-level interventions to reduce alcohol-related harm: An overview of systematic reviews. Preventive Medicine 2013; 57: 278-296<br>International Center for Alcohol Policies. Determinants of drinking. ICAP Health Issues (Online). Available: http://www.icap.org/linkclick.aspx?fileticket=oHFi97riJos%3d&tabid=243, 2009.<br>**Economic data, for example:**<br>Donnelly N, Scott L, Poyton S, et al. Estimating the short-term cost of police time spent dealing with alcohol-related crime in NSW. Monograph Series No 25. Tasmania: National Drug Law Enforcement Research Fund; An Initiative of the National Drug Strategy, 2007.
