# Supplementary material for: Harnessing advances in computer simulation to inform policy and planning to reduce alcohol-related harms
Source: Int J Public Health. 2017 Oct 19;63(4):537–46. doi: 10.1007/s00038-017-1041-y (PMC5938302; doi:10.1007/s00038-017-1041-y)
Supplement: Supplementary file 4 — Supplementary material 4 (PDF 433 kb) [file 38_2017_1041_MOESM4_ESM.pdf]

Title: ***Harnessing advances in computer simulation to inform policy and planning to reduce alcohol-related harms***

Journal: ***International Journal of Public Health***

Authors: Jo-An Atkinson[a,b,c], Dylan Knowles, John Wiggers, Michael Livingston, Robin Room, Ante Prodan, Geoff McDonnell, Eloise O'Donnell, Sandra Jones, Paul S Haber, David Muscatello, Nadine Ezard, Nghi Phung, Louise Freebairn, Devon Indig, Lucie Rychetnik, Jaithri Ananthapavan, and Sonia Wutzke, on behalf of the alcohol modelling consortium.

Affiliations of corresponding author:

[a] The Australian Prevention Partnership Centre, Sax Institute, Sydney, Australia

[b] Sydney Medical School, University of Sydney, Australia

[c] Corresponding author: The Australian Prevention Partnership Centre, Sax Institute, PO Box K617, Haymarket, NSW, 1240, Australia, Jo-An.Atkinson@saxinstitute.org.au; Ph: +61 2 9188 9537; Fax: +61 2 9188 9501

Online Resource 4: Data processing method (NSW alcohol model)

Data Processing Methodology

12 independent simulation runs for each scenario

Approximately 7GB of data for a 11 years period for each scenario, 250GB of data in total.

Are 12 simulation runs sufficient?

It is assumed that 12 simulation runs are sufficient based on the fact that each simulation comprises of approximately 3.6 million individuals. This assumption is validated by comparing the results of 12 and 24 independent simulation runs for one of the scenarios and then obtaining the difference between sample means. The results, provided in the table bellow, are well within the margin of error of the 12 simulation runs results.

	Diff Means %	Diff MERR %
ED Presentation	-0.05	0.05
Harms Generated	0.08	0.24
Hospitalisations	0.00	-0.06

The results processed in R*

(a language for statistical computing and graphics)

- The data for the first year is not considered to allow for the simulation to stabilise.
- The datasets from the each simulation run are processed and descriptive statistics for all variables is evaluated as a part of the simulation calibration and validation process. This step is iterated until satisfactory accuracy is achieved without overfitting.
- Once the simulation is validated and calibrated the descriptive statistics are generated across all runs for all variables captured from the simulations cumulatively and monthly for the 5-year period. This includes 48 tables and 22 figures for each scenario.
- The tables containing a comparison of simulation results between baseline and other scenarios are created. These tables provide the difference in the outcomes of the scenarios expressed as a difference in the mean of two independent samples for all captured variables for the same population.

*R Core Team (2012). R: A language and environment for statistical computing. R Foundation for Statistical Computing, Vienna, Austria. ISBN 3-900051-07-0, URL http://www.R-project.org/
